# Supplementary material for: Self-actualization and B-values: Development and validation of two instruments in the Brazilian context
Source: PLoS One. 2024 Jun 7;19(6):e0302322. doi: 10.1371/journal.pone.0302322 (PMC11161018; doi:10.1371/journal.pone.0302322)
Supplement: S1 File — (ZIP) [file pone.0302322.s001.zip › Instruments/00 - B-Values Inventory (BVI) (English Version).docx]

**B-Values Inventory (BVI)**

**INSTRUCTIONS**. Below is a description of some characteristics that may or may not describe you. Please choose one of the numbers on the scale below that best expresses you:

| Doesn’t describe me at all |  | Neutral |  | Describe me completely |
| --- | --- | --- | --- | --- |
| 1 | 2 | 3 | 4 | 5 |

**Generally, I feel like I am a person who prioritizes:**

| Truth. True person who avoids telling lies. | [ ] |
| --- | --- |
| Playfulness. Cheerful person who prioritizes good humor. | [ ] |
| Aliveness. Person full of energy, with vigor and spontaneity. | [ ] |
| Perfection. Meticulous person who strives for excellence and perfection. | [ ] |
| Goodness. Kind person who prioritizes altruism and benevolence. | [ ] |
| Justice. Person with a sense of justice who prioritizes order and merit. | [ ] |
| Beauty. Person inclined towards art who prioritizes beauty and aesthetics. | [ ] |
| Simplicity. Person inclined to simplicity, who cares about the simple things in life. | [ ] |
| Richness. Complete and fulfilled person who seeks the height of its fullness. | [ ] |
| Singularity. Original person who seeks to be unique and exclusive in its individuality. | [ ] |
| Effortlessness. Thrifty person who aims to accomplish important tasks with efficiency and low effort. | [ ] |
| Completion. Accomplished person who undertakes significant tasks and fulfilling one’s destiny. | [ ] |
| Self-sufficiency. Self-sufficient person who seeks to have autonomy and independence in what does. | [ ] |
| Wholeness. A person of integrity who prioritizes seriousness, honesty and dignity. | [ ] |
