# Supplementary material for: Self-actualization and B-values: Development and validation of two instruments in the Brazilian context
Source: PLoS One. 2024 Jun 7;19(6):e0302322. doi: 10.1371/journal.pone.0302322 (PMC11161018; doi:10.1371/journal.pone.0302322)
Supplement: S1 File — (ZIP) [file pone.0302322.s001.zip › Instruments/00 - Escala de Atributos da Autorrealizaç╞o (EAAr) (Portuguese Version).docx]

**Escala de Atributos da Autorrealização (EAAr)**

INSTRUÇÕES. Pense no seu trabalho. Em seguida, leia as afirmações e pontue o quanto elas lhe descrevem:


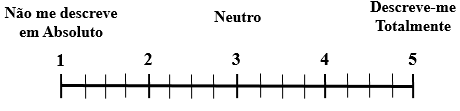


| Quando estou trabalhando, estou me divertindo. | [ ] |
| --- | --- |
| Acredito que meu trabalho pode mudar o mundo ou os seus paradigmas. | [ ] |
| Busco a excelência profissional (técnica) no meu trabalho. | [ ] |
| Vejo beleza no trabalho que realizo. | [ ] |
| Meu trabalho é intrínseco à minha própria natureza. | [ ] |
| Esqueço-me dos problemas cotidianos quando estou trabalhando. | [ ] |
| Sei qual trabalho/profissão me realiza pessoalmente. | [ ] |
| As conquistas relacionadas ao meu trabalho são uma fonte de felicidade. | [ ] |
| Gosto de falar sobre o que eu estou trabalhando com outras pessoas. | [ ] |
| Sinto-me alegremente ansioso para realizar o meu trabalho. | [ ] |
| Sinto-me orgulhoso do meu trabalho. | [ ] |
| Às vezes, sinto-me eufórico quando estou realizando o meu trabalho. | [ ] |
| Sei que as outras pessoas me reconhecem pelo que eu faço. | [ ] |
| Não me importo de trabalhar algumas horas a mais em meu trabalho. | [ ] |
| Dentre outras coisas, o meu trabalho traz sentido à minha vida. | [ ] |
| Meu trabalho mostra quem eu realmente sou | [ ] |
| Realizar o meu trabalho me faz bem. | [ ] |
| Minha vida profissional tem propósito. | [ ] |
| Meu trabalho é parte muito importante da minha vida. | [ ] |
| Procuro ser tudo o que sou capaz de ser no meu trabalho. | [ ] |
| Busco a excelência pessoal no meu trabalho. |  |
| Procuro me doar ao máximo ao meu trabalho, explorando todo meu potencial. | [ ] |
| Sinto-me realizado pelo que eu faço. | [ ] |
| Tenho consciência do meu potencial. | [ ] |
| Busco conhecimento para ser cada dia melhor. | [ ] |
| Ao finalizar o meu trabalho, sinto que poderia realizar essa mesma atividade por muitos anos, pela satisfação que me traz. | [ ] |
| Sinto-me realizado com o que me tornei profissionalmente. | [ ] |
| Procuro evoluir e me desenvolver. | [ ] |
| Busco sempre a excelência de mim mesmo. | [ ] |
| Sinto-me espiritualmente ligado ao meu trabalho. | [ ] |
| Sou apaixonado pelo que eu faço. | [ ] |
| Ao finalizar o meu trabalho, sinto que fiz o meu papel no mundo. | [ ] |
| Meu desempenho profissional aumenta a minha autoestima. | [ ] |
| Minha profissão está relacionada aos meus talentos. | [ ] |
| Quando eu estou trabalhando eu perco a noção do tempo. | [ ] |
