# Supplementary material for: Self-actualization and B-values: Development and validation of two instruments in the Brazilian context
Source: PLoS One. 2024 Jun 7;19(6):e0302322. doi: 10.1371/journal.pone.0302322 (PMC11161018; doi:10.1371/journal.pone.0302322)
Supplement: S1 File — (ZIP) [file pone.0302322.s001.zip › Instruments/00 - Inventário de Valores-B (IV-B) (Portuguese Version).docx]

**Escala de Características dos Valores-B**

**INSTRUÇÕES**. A seguir encontram-se a descrição de algumas características que podem ou não lhe descrever. Por favor, escolha um dos números na escala abaixo que melhor expresse você:


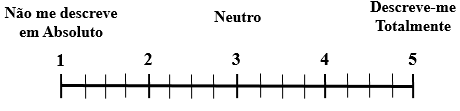


**Geralmente, sinto que sou uma pessoa:**

| Verdadeira, que evita contar mentiras. | [ ] |
| --- | --- |
| Alegre, que prioriza o bom humor. | [ ] |
| Cheia de energia, com vigor e espontaneidade. | [ ] |
| Minuciosa, que busca a excelência e a perfeição. | [ ] |
| Bondosa, que prioriza o altruísmo e a benevolência. | [ ] |
| Com senso de justiça, que prioriza a ordem e o merecimento. | [ ] |
| Inclinada à arte, que prioriza a beleza e a estética. | [ ] |
| Inclinada à simplicidade, que se importa com as coisas simples da vida. | [ ] |
| Completa, preenchida, que busca o auge da sua plenitude. | [ ] |
| Original, que busca ser único e exclusivo em sua individualidade. | [ ] |
| Parcimoniosa, que procura fazer coisas importantes com eficácia e baixo esforço. | [ ] |
| Realizada, que desempenha coisas importantes e cumpre o próprio destino. | [ ] |
| Autossuficiente, que procuro ter autonomia e independência no que faço. | [ ] |
| Íntegra, que prioriza a seriedade, a lisura e a dignidade. | [ ] |
