# Supplementary material for: Self-actualization and B-values: Development and validation of two instruments in the Brazilian context
Source: PLoS One. 2024 Jun 7;19(6):e0302322. doi: 10.1371/journal.pone.0302322 (PMC11161018; doi:10.1371/journal.pone.0302322)
Supplement: S1 File — (ZIP) [file pone.0302322.s001.zip › Instruments/00 - Self-Actualization Attributes Scale (SAAS) (English Version).docx]

**Self-Actualization Attributes Scale (SAAS)**

**INSTRUCTIONS**. Think about your job. Then, read the statements and rate how much they describe you:

| Doesn’t describe me at all |  | Neutral |  | Describe me completely |
| --- | --- | --- | --- | --- |
| 1 | 2 | 3 | 4 | 5 |

| 2. When working, I am having fun. | [ ] |
| --- | --- |
| 3. I believe my job can change the world or its paradigms. | [ ] |
| 4. I pursue professional (technical) excellence in my job. | [ ] |
| 6. I see beauty in the job I perform. | [ ] |
| 7. My job is intrinsic to my own nature. | [ ] |
| 8. I forget about everyday problems when I am working. | [ ] |
| 9. I know which job/profession personally fulfills me. | [ ] |
| 10. Achievements related to my job are a source of happiness. | [ ] |
| 11. I enjoy discussing what I am working on with others. | [ ] |
| 12. I eagerly look forward to carrying out my job. | [ ] |
| 13. I feel proud of my job. | [ ] |
| 14. At times, I feel euphoric when engaged in my work. | [ ] |
| 15. I know that others recognize me for what I do in my job. | [ ] |
| 16. I don’t mind working extra hours in my job. | [ ] |
| 17. Among other things, my job gives meaning to my life. | [ ] |
| 18. My job reflects who I truly am. | [ ] |
| 19. Executing my job brings me well-being. | [ ] |
| 20. My professional life has purpose. | [ ] |
| 21. My job is a very important part of my life. | [ ] |
| 22. I aim to be everything I am capable of being in my work. | [ ] |
| 23. I seek personal excellence in my job. |  |
| 24. I strive to dedicate myself fully to my job, exploring my full potential. | [ ] |
| 25. I feel fulfilled by what I do. | [ ] |
| 26. I am aware of my potential. | [ ] |
| 27. I seek knowledge to improve myself every day. | [ ] |
| 28. Upon completing my job, I feel that I could continue this activity for many years due to the satisfaction it brings. | [ ] |
| 29. I feel satisfied with what I have become professionally. | [ ] |
| 30. I aim to evolve and develop myself. | [ ] |
| 32. I consistently strive for excellence in myself. | [ ] |
| 34. I feel spiritually connected to my work. | [ ] |
| 35. I am passionate about what I do. | [ ] |
| 36. Upon completing my work, I feel that I have played my role in the world. | [ ] |
| 37. My professional performance enhances my self-esteem. | [ ] |
| 39. My profession is aligned with my talents. | [ ] |
| 40. When I am working, I lose track of time. | [ ] |
