# Supplementary material for: Self-actualization and B-values: Development and validation of two instruments in the Brazilian context
Source: PLoS One. 2024 Jun 7;19(6):e0302322. doi: 10.1371/journal.pone.0302322 (PMC11161018; doi:10.1371/journal.pone.0302322)
Supplement: Attachment — Submitted filename: Response to Reviewers.pdf [file pone.0302322.s003.pdf]

Response to Reviewers

Dear Editor,

We would like to inform you that we reviewed all the points indicated in the general editorial review. And all the points indicated by the reviewers have been taken into account. We have reviewed each point of correction, adequacy, or insertion. We would like to point out that some points were only partially resolved; however, these points were duly and thoroughly justified, as shown in the table below.

We remain at your disposal for any further revisions.

Best regards,

Prof. Gustavo Henrique Silva de Souza
Prof. Jorge Artur Peçanha de Miranda Coelho
Prof. Nilton Cesar Lima
Prof. Germano Gabriel Lima Esteves
Prof. Fernanda Cristina Barbosa Pereira Queiroz
Prof. Yuri Bento Marques

**Reviewer's comment**	**Modification made by the authors**
**Reviewer #1**	
In general, the authors divided their manuscript into numerous short parts. Several parts should be merged into longer paragraphs. A paragraph should include at least three sentences.	Ok. The text was revised, and some paragraphs were adjusted.
Please re-struct this manuscript following the normal structure of an article, including Introduction, Methods, Results, Discussion, and Conclusion.	Ok. The structure was revised.<br><br>Due to the type of study presented, it is very complex to rigidly follow the "Introduction, Methods, Results, Discussion and Conclusions" structure. This structure disregards the fact that the study has different stages and requires a more robust and detailed theoretical foundation, as it deals with a complex psychological construct. In this sense, the requested structure was followed, but sub-sections were added, in line with other articles published in Plos One, for example, the article "Testing the cultural-invariance hypothesis: A global analysis of the relationship between scientific knowledge and attitudes to science", follows a structure of "Introduction, Background and relevant literature, Data and measures, Analysis strategy, Results, and Discussion".
Why did the authors choose a large number of professors (about two-thirds) for this survey? This group is only a minority of the population. Who will be the target	As explained in the "Procedures / Participants Recruitment" sub-section, the sampling procedure was by convenience. In this sense, participants were recruited by e-mail, and since all the authors of this study are teachers, our main contacts are also teachers.

population of these instruments?	A sentence was added to align the teaching profession with the research objectives.
Please add the information involving the response rate of participants.	Ok. Information entered as requested in the subsection “Procedures / Participants Recruitment”.
Please explain why the authors used IPS and BVQ questionnaires in the Methods section.	The aim of this study deals specifically with innovative behavior and human values, as there are indications in the literature of a relationship between the constructs.<br><br>The IPS and BVQ were used to test this relationship, as explained in the subsection “Data Analysis”.
The authors should not cite references in the Results section.	We partially agree with the reviewer. The references indicated in the text of the results have been inserted to provide a basis for interpreting the data analysis. To improve understanding, phrases such as “as recommended in the literature” or “see parameters from the literature” have been added. Thus, we believe that mentioning the theoretical basis for interpreting the results makes the article more robust.
**Reviewer #2**	
Title: The title should emphasise “the development and validation of the instrument” instead of the “measurement of the construct”.	Ok. Partially attended to. Although the study specifically addresses the construction and validation of the 2 instruments, the central focus is the measurement of 2 important constructs. In this sense, we believe that starting the title mentioning the 2 constructs makes the article more visible and attracts more attention. We changed it to “Self-actualization and b-values: Development and validation of two instruments in the Brazilian context”.
Aim:<br>• The wording of the aim in the abstract is rather unclear. Please revise. Suggestion: Developing, validating, and cross-verifying measures for self-actualization attributes and B-values, focusing on job context and theoretical congruence with innovative behaviour and human values related to the self-actualization construct (suprapersonal subfunction).<br>• The wording of the aim in the introduction needs to be shortened. Please make it concise and consistent with the aim stated in the abstract.	Ok. We have changed the wording of the study objective as recommended.
Abstract:<br>The abstract stated that the method encompassed: “The proposed instrument underwent content and semantic validity assessments, followed by verification of factor validity and internal consistency.	Ok. We have rewritten much of the Abstract to suit the suggestions.

Additional evidence of convergent validity was also examined:” No information in the method on the constructs that instrument were cross-validated in the convergent validity and no information on the results regarding the findings related to content and validity assessment, factor analysis and internal consistency reliability<br>Suggestion: Rewrote the abstract to accommodate the missing information within the word limit.	
Introduction<br>The introduction (heading 1) and heading 2 is rather hard to follow, please make it more concise and simple for lay readers<br>Suggestion: Combine and rewrite headings 1 and 2 within the introduction section, following the IMRAD flow.	Ok. We have made changes to improve comprehension.
Method<br>Suggestion: Following the IMRAD flow, please put Heading 3 and 4 under the method section.	Ok. We have placed the instrument construction subsections in the “methods” section.
Results<br>Please put the findings of the content and semantic assessment in the results section instead of in the method section.	Ok. We partially agree with the reviewer. We believe that the results of the semantic and content assessment can be kept in the methods section, as they are preliminary analyses of the process of writing. There is no detriment to the intelligibility of the study and it facilitates a chronological understanding of the research process.
Overall: Please add research implications and recommendations regarding practical and clinical application.	Ok. We agree with the reviewer. Texts have been included in response to this suggestion.
**Reviewer #3**	
Ideally, if possible, I would have expected some additional criteria validity analysis, assessing the extent to which a person's performance on these questionnaires conforms to external criteria or standards that are relevant to the construct the test is intended to measure, such as burnout, stress, or some general mental health. If the authors have something like that, I suggest including it. Nevertheless, this would be a minor suggestion.	We fully agree with the reviewer. We should have provided additional criterion validity analyses, but for this study it is no longer possible to include new constructs for analysis. Tests for burnout, stress, or some general mental health were not applied in the moment of the research (between June 29 and August 31, 2020).
**Reviewer #4**	
Purpose and results are not	The text was revised, and some corrections were made

adequately presented, which results in the reader not being able to extract the focus of the study from it, and the authors are asked to add clarity.	in the article.
It is recommended that authors use subheadings to make the abstract section clearer, as in the case of “Background”, “Objective”, “Methods”, “Results”, “Conclusion”.	Ok. The structure was revised, as recommended.
Overall: Abstracts require a brief summary of the article's background, aims, methods, results and conclusions. The current abstract does not provide a good overview. Please refer to abstracts of high quality articles and rewrite the abstract.	Ok. We agree with the reviewer. The Abstract has been readjusted.
Introduction<br>Line34-37 Page 1: Please describe each requirement specifically..	Ok. Revised as requested. Additional text has been added.
Line52-56 Page 1: Please elaborate further. The current description does not give me a clear picture of this part of your research.	Ok. Revised as requested. Additional text has been added to improve understanding.
Line229-233 Page 6:<br>1) Whether there were inclusion or exclusion criteria for the study population, and if so, please state this in the article.	Ok. Revised as requested. Additional information on inclusion or exclusion criteria for the study population has been included in the article.
2) How was the sample size determined, give the calculation or supporting references.	The non-probabilistic survey method automatically assumes that there is no sample size calculation. An additional reference has been included.
3) Is 621 the final study sample?	Yes. 621 is the final sample of the study. Additional information on the samples has been included in the article.
4) Could the authors please clarify, as well as whether there is missing data and how to deal with missing data.	Ok. Revised as requested. Additional information on missing data has been included in the article.
5) It is recommended that a table of demographic characteristics be created.	We believe that creating a table with demographic information is unnecessary for understanding the sample and interpreting the results. There are no variables other than those already presented.
6) “From 25 different 229 professions”, please indicate what each of the 25 occupations is?	We believe that mentioning each of the 25 professions is unnecessary, especially since the manuscript is almost 8,000 words long. We have added the second most common profession, administrators (5%). The percentage of other professions is too small to mention.
Line 235-238 Page 6: Please describe the process in detail, e.g. what is the source of the participants? Are participants paid	Ok. Revised as requested.

accordingly, etc.?	
Line 286-287 Page 7: " v12.01.02 286 ", "v" is an abbreviation, used here for the first time, which should be interpreted (version), Please revise it.	Ok. Revised as requested.
Line 319 Page 8: Authors are asked to double-check the parts of the text that require italics, such as χ2.	Ok. Revised as requested.
Line 355 Page 10:<br>1) The decimal points are not harmonized.<br>2) The author needs to check that all abbreviations are explained e.g. CFI, TLI.<br>3) (χ2) needs to be italicized.	Ok. Revised as requested. CFI and TLI were explained in subsection "Data Analysis".
Line 367 Page 10: "(χ2(91) 367= 3052.8; p = 0.00)", χ2 needs to be italicized.	Ok. Revised as requested.
Line 379 Page 11: "(H =0.87)", Spaces are required before and after the equal sign.	Ok. Corrected as requested.
Line 387 Page 11:<br>1) The decimal points are not harmonized.<br>2) The CFI TLI is not explained.<br>3) (χ2) needs to be italicized.	Ok. Revised as requested. CFI and TLI were explained in subsection "Data Analysis".
Conclusion<br>Overall: For an article, the conclusion is a necessary demonstration of important findings and the value of the research, which the authors are invited to add.	Ok. We agree with the reviewer. Revised as requested. The conclusion section was inserted and merged with the section of Limitations and Future Research.
Other recommendations:<br>Overall: Give the source of funding and the role of the funders for the present study.	Ok. The PLOS ONE guidelines state that the source of funding information should be entered into the system and not in the manuscript. We have inserted the source of funding in the system, as recommended.
The author does not seem to be very good at essay writing and many of the basic requirements are not met. There are a number of problems with this essay: 1) punctuation conforms to incorrect use, 2) much of it is not detailed enough, and 3) the current analysis is too simplistic to explain the purpose of the study.	Ok. Revised as requested. We made some changes and revised the entire text of the manuscript.
