# Supplementary material for: Self-actualization and B-values: Development and validation of two instruments in the Brazilian context
Source: PLoS One. 2024 Jun 7;19(6):e0302322. doi: 10.1371/journal.pone.0302322 (PMC11161018; doi:10.1371/journal.pone.0302322)
Supplement: Attachment — Submitted filename: PLOS one General comment.docx [file pone.0302322.s002.docx]

General comment:

The article is well-written, however, some improvement is required to increase the clarity

Specific comment :

1. Title: The title should emphasise “the development and validation of the instrument” instead of the “measurement of the construct”.
2. Aim:

- The wording of the aim in the abstract is rather unclear. Please revise. Suggestion: Developing, validating, and cross-verifying measures for self-actualization attributes and B-values, focusing on job context and theoretical congruence with innovative behaviour and human values related to the self-actualization construct (suprapersonal subfunction).
- The wording of the aim in the introduction needs to be shortened. Please make it concise and consistent with the aim stated in the abstract.

3. Abstract:

The abstract stated that the method encompassed: *“The proposed instrument underwent content and semantic validity assessments, followed by verification of factor validity and internal consistency. Additional evidence of convergent validity was also examined:”* No information in the method on the constructs that instrument were cross-validated in the convergent validity and no information on the results regarding the findings related to content and validity assessment, factor analysis and internal consistency reliability

Suggestion: Rewrote the abstract to accommodate the missing information within the word limit

4. Introduction

The introduction (heading 1 ) and heading 2 is rather hard to follow, please make it more concise and simple for lay readers

Suggestion: Combine and rewrite headings 1 and 2 within the introduction section, following the IMRAD flow

5. Method

Suggestion: Following the IMRAD flow, please put Heading 3 and 4 under the method section

6. Results

Please put the findings of the content and semantic assessment in the results section instead of in the method section

Overall: Please add research implications and recommendations regarding practical and clinical application
